# Multiple Signaling Pathways Coordinate to Induce a Threshold Response in a Chordate Embryo

**DOI:** 10.1371/journal.pgen.1003818

**Published:** 2013-10-03

**Authors:** Naoyuki Ohta, Yutaka Satou

**Affiliations:** Department of Zoology, Graduate School of Science, Kyoto University, Sakyo, Kyoto, Japan; New York University, United States of America

## Abstract

In animal development, secreted signaling molecules evoke all-or-none threshold responses of target gene transcription to specify cell fates. In the chordate *Ciona intestinalis*, the neural markers *Otx* and *Nodal* are induced at early embryonic stages by Fgf9/16/20 signaling. Here we show that three additional signaling molecules act negatively to generate a sharp expression boundary for neural genes. EphrinA signaling antagonizes FGF signaling by inhibiting ERK phosphorylation more strongly in epidermal cells than in neural cells, which accentuates differences in the strength of ERK activation. However, even weakly activated ERK activates *Otx* and *Nodal* transcription occasionally, probably because of the inherently stochastic nature of signal transduction processes and binding of transcription factors to target sequences. This occasional and undesirable activation of neural genes by weak residual ERK activity is directly repressed by Smad transcription factors activated by Admp and Gdf1/3-like signaling, further sharpening the differential responses of cells to FGF signaling. Thus, these signaling pathways coordinate to evoke a threshold response that delineates a sharp expression boundary.

## Author Summary

Graded signals often provide positional information to organize gene expression in animal embryos. In the simplest cases, graded signals are translated into all-or-none threshold responses. However, recent studies have shown that signal transduction processes and binding of transcription factors to target sequences are inherently stochastic. This means that even weak activating signaling might activate target genes stochastically. However, the precise mechanism, by which this stochastic undesirable activation is avoided, is still largely unknown. In the embryo of a simple chordate, *Ciona intestinalis*, FGF signaling is known to induce neural fate. In the present study, we demonstrate that three additional signaling molecules cooperate to evoke a threshold response for specification of neural fate. First, EphrinA signaling inhibits FGF signaling by attenuating ERK phosphorylation, accentuating differences in the strength of ERK activation. However, even weak ERK activity occasionally turns on the neural genes. This occasional undesirable activation of the neural genes is turned off by Admp and Gdf1/3 signaling through Smad transcription factors. Thus, these two qualitatively different negative regulatory mechanisms evoke an all-or-none threshold response to specify neural fate.

## Introduction

In animal development, secreted signaling molecules often elicit the production of multiple cellular identities by controlling the activity of transcription factors. Molecular gradients can produce differential responses in identical cells [1], [2]. For example, in *Drosophila* syncytium embryos, a concentration gradient of the transcription factor Bicoid specifies the anterior-posterior axis [3], [4]. In the vertebrate neural tube, a gradient of the secreted signaling molecule Sonic Hedgehog is responsible for defining five distinct neural progenitor domains [5]–[7]. Translation of a graded distribution of molecules into sharp gene expression boundaries is central to many developmental processes, but apart from a few cases, the molecular mechanisms underlying this process are not yet fully understood. Especially, even a weak signal can potentially activate transcription of target genes due to the inherently stochastic nature of signal transduction processes and binding of transcription factors to target sequences [8]. How is such weak undesirable activation blocked in animal embryos?

**Figure 1:**
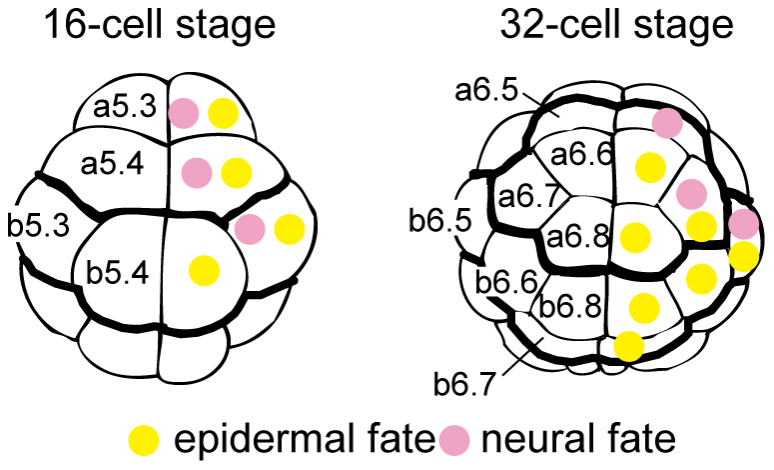
Fate maps of the animal hemisphere of 16- and 32-cell embryos. Epidermal and neural fates are indicated in the right halves by yellow and pink circles, respectively. Blastomere names are indicated in the left halves. The anterior-posterior boundaries and the animal-vegetal boundaries are shown as thick lines.

Cells in the animal hemisphere of ascidian embryos (*Ciona intestinalis*) give rise to both epidermal and neural cells (Figure 1). At the 32-cell stage, an earliest neural marker gene, *Otx*, begins to be expressed in a pair of anterior animal cells (a6.5) and a pair of posterior animal cells (b6.5), and *Nodal* expression also begins in b6.5 (Figure S1A and S1B) [9]–[11]. Some embryos also express *Otx* in a6.7 [12], indicating that *Otx* expression in a6.7 is not tightly regulated. In the present study, we disregarded this cryptic expression unless otherwise noted. The remaining animal cells are all restricted to epidermal fate. In addition, *Otx* and *Nodal* are expressed in vegetal cells (Figure S1A and S1B). *Otx* is required for subsequent expression of neural genes [13], and ectopic *Nodal* expression in non-neural ectodermal cells results in embryonic patterning defects [14].

At the 16-cell stage, all ectodermal cells express the same set of regulatory genes, except for *FoxA-a*, which is expressed in anterior but not posterior cells [10] (Figure S1C). Even though *FoxA-a* activates the anterior fate, some other instructive mechanism likely functions to induce neural fate. However, no asymmetric localization of maternal mRNA has been detected in the animal hemisphere in spite of extensive efforts to identify such a molecule. In addition, a cell dissociation experiment indicated that cell-cell interactions are required for specification of the neural fate [9]. Therefore, it is likely that neural fate is specified primarily by cell-cell interactions.

It is possible that maternally provided signaling molecules and mRNAs encoding signaling molecules play a role in the specification of neural fate, even if they are distributed evenly within the embryo. However, it is more likely that signaling molecules expressed from the zygotic genome of specific cells play a more important role. Our previous study [10] showed that only five signaling ligand genes are zygotically expressed at the 16-cell stage, one stage earlier than the 32-cell stage when *Otx* and *Nodal* expression begins (Figure S1D–H). *Fgf9/16/20* is expressed in all of the vegetal cells except for the most posterior ones [15], [16], *EphrinA-d* is expressed in the entire animal hemisphere, *Wnt-NAe* (a Wnt ligand gene whose phylogenetic position is unclear) and *Admp* are expressed in posterior vegetal cells (B5.1), and *Gdf1/3-like* (or *orphan Tgfβ-1*), is expressed in the entire animal hemisphere.

Among the ectodermal cells of the 32-cell embryo, cells with neural fate have a larger area of surface contact with FGF-expressing vegetal cells and are accordingly expected to be exposed to stronger FGF signaling [12]. This results in activation of maternal GATA and Ets transcription factors, which in turn directly activate *Otx* expression [16]. *Nodal* is similarly activated [17], but only in b6.5. However, because non-neural ectodermal cells also contact vegetal cells expressing *Fgf9/16/20*, it is very likely that these cells are exposed to weak FGF signaling. Due to the inherently stochastic nature [8], even weak FGF signaling might activate *Otx* and *Nodal* enhancers. In the present study, we show that weak FGF signaling indeed activates *Otx* and *Nodal* expression, and that EphrinA signaling amplifies the difference in ERK phosphorylation levels induced by differing strength of FGF signaling. Moreover, the occasional activation of *Otx* and *Nodal* by residual weak ERK activity is repressed by Admp/Gdf1/3-like signaling. Thus, FGF, Ephrin, and Admp/Gdf1/3-like signaling cooperate to evoke a threshold response to establish neural fate.

## Results

### *Nodal* is repressed in the anterior neural cells by *FoxA-a*

**Figure 2:**
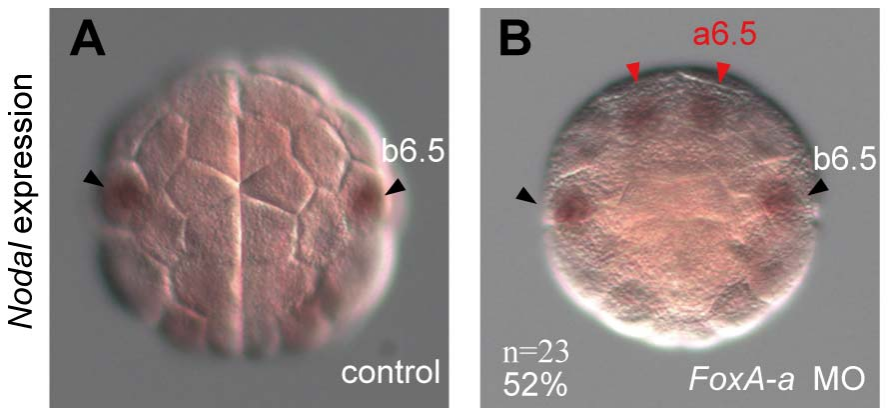
*FoxA-a* negatively regulates *Nodal* expression in the anterior neural cells. (A, B) Expression of *Nodal* in (A) a control embryo and (B) a *FoxA-a* morphant embryo. *Nodal* is expressed ectopically in a6.5 at the 32-cell stage (red arrowheads) in the *FoxA-a* morphant.

Our previous comprehensive screen [10] showed that *FoxA-a* is the only regulatory gene that are expressed differently between the a- and b-line cells. FoxA-a directly activates ectodermal genes in anterior cells at later stages [18] and represses *Nodal* at the early gastrula stage [13]. Therefore, we first examined whether *FoxA-a* similarly represses *Nodal* at the 32-cell stage. In embryos injected with an antisense morpholino oligonucleotide (MO) for *FoxA-a*, *Nodal* expression was indeed expressed ectopically in a6.5 at the 32-cell stage (Figure 2), indicating that *FoxA-a* normally suppresses *Nodal* expression in anterior cells.

### Multiple signaling pathways coordinate to control expression of *Otx* and *Nodal*

As we described in the Introduction section, neural fate is probably specified primarily by cell-cell interactions. To understand the mechanisms that activate *Otx* and *Nodal* specifically in the neural lineage, we examined the functions of the five signaling ligand genes that are expressed at the 16-cell stage.

**Figure 3:**
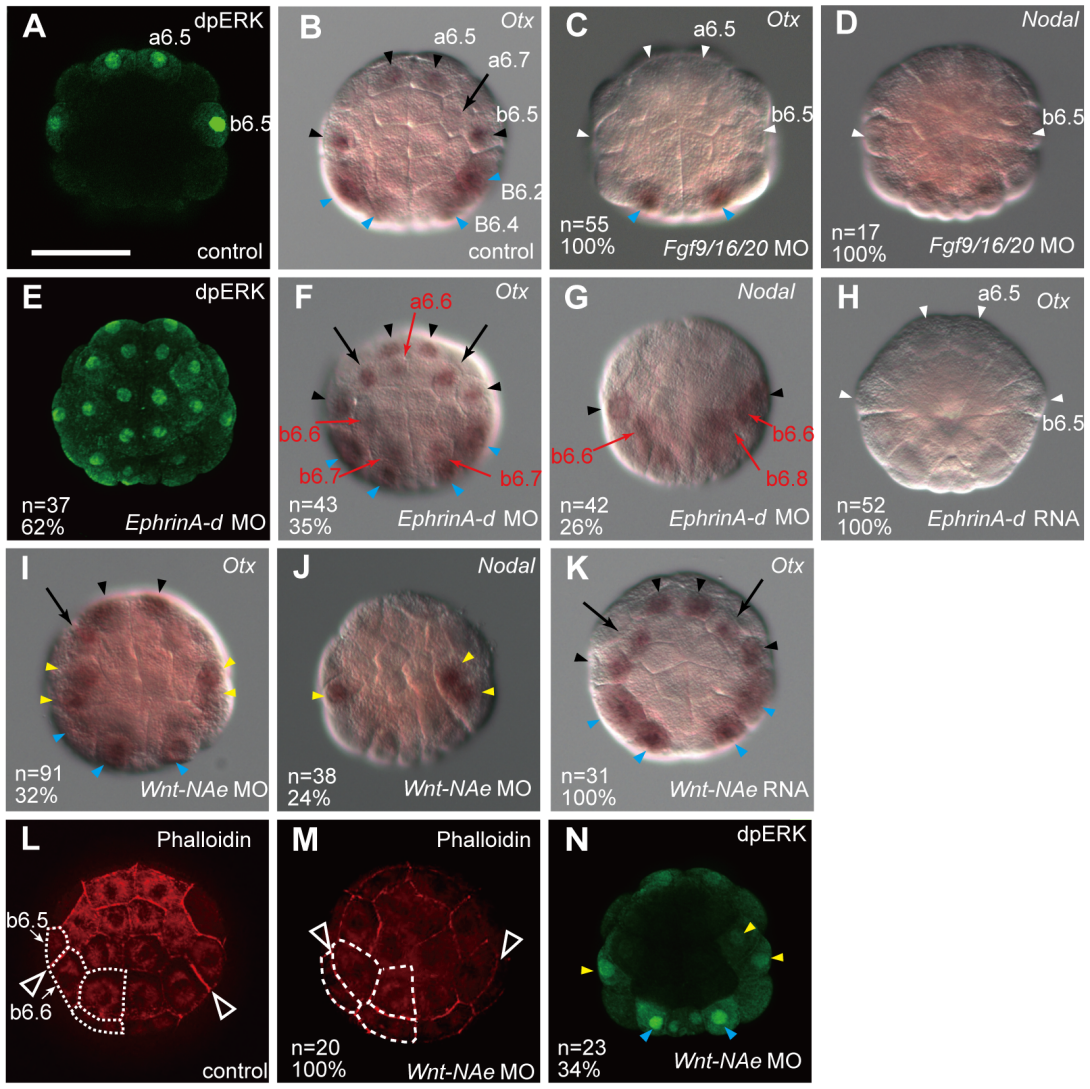
Fgf, Ephrin and Wnt signaling cooperatively regulates neural fate. (A, E, N) Immunostaining of dpERK in (A) a control embryo, (E) an *EphrinA-d* morphant, and (N) a *Wnt-NAe* morphant at the 32-cell stage. (B–D, F–K), Expression of (B, C, F, H, I, K) *Otx* and (D, G, J) *Nodal* in (B) a control embryo, and in 32-cell embryos injected with MOs for (C, D) *Fgf9/16/20*, (F, G) *EphrinA-d*, and (I, J) *Wnt-NAe*, and RNAs of (H) *EphrinA-d* and (K) *Wnt-NAe*. Loss of expression is indicated by white arrowheads, while ectopic expression is indicated by red arrows. Expression in a6.5 and b6.5 is indicated by black arrowheads, and cryptic expression in a6.7 is indicated by black arrows. Expression in vegetal cells, which was not analyzed in the present study, is indicated by blue arrowheads. Yellow arrowheads in (I–K) indicate the daughter cells of b5.3 (b6.5 and b6.6) in *Wnt-NAe* morphants. The effect of FGF signaling on neural marker expression shown in (A–D) was shown in previous studies [9], 16,17,19. (L, M) Phalloidin staining to highlight the cell membrane in (L) a control embryo and (M) a *Wnt-NAe* morphant at the 32-cell stage. The posterior animal (b-line) cells on the left side are outlined by white dotted lines. The boundaries of the b6.5 and b6.6 sister cells are shown by arrowheads. All embryos are shown in an animal view. The scale bar in (A) represents 100 µm.

We first confirmed the effect of FGF signaling on neural marker expression. As previously shown [9], [16], [17], [19], responsiveness to FGF signaling, as indicated by activated ERK (dpERK), was observed in a6.5 and b6.5 in normal 32-cell embryos (Figure 3A), and expression of *Otx* and *Nodal* was absent from the animal hemisphere in *Fgf9/16/20* morphants (Figure 3B–D; Tables S1 and S2). On the other hand, overexpression of *Fgf9/16/20* by synthetic RNA microinjection into fertilized eggs and one posterior animal cell of 8-cell embryos resulted in ectopic expression of *Otx* (Figure S2A and S2B) [16]. Thus, FGF signaling activates *Otx* and *Nodal* expression via ERK activation.

As previously shown in later stage embryos [20]–[22] and in vertebrates [23], *EphrinA-d* attenuated ERK phosphorylation in 32-cell embryos, as indicated by the fact that dpERK immunostaining was observed ectopically in all of the animal blastomeres of *EphrinA-d* morphants (Figure 3E). *Otx* was expressed ectopically in animal cells in *EphrinA-d* morphants, and *Nodal* was expressed ectopically in posterior animal cells in these morphants (Figure 3F and 3G; Tables S1 and S2). Conversely, overexpression of *EphrinA-d* resulted in complete loss of *Otx* expression (Figure 3H). Thus, all of the animal cells indeed receive FGF signaling, and *EphrinA-d* appears to modulate FGF signaling by inhibiting ERK phosphorylation, generating clear differences in the strength of ERK activation.

In a previous study [12], “3D-virtual embryos” were reconstructed and the surface contacts of cells with their surrounding cells were calculated. This work showed that a6.5 and b6.5 have the greatest surface contacts with *Fgf9/16/20*-expressing cells and suggested that differences in the contact area of competent cells are important for *Otx* expression in neural cells [12]. Our calculation using this tool indicated that a6.5 and b6.5 have the least surface contact with *EphrinA-d*-expressing cells (Figure S3). Therefore, a6.5 and b6.5 are likely subject to the lowest levels of inhibitory signals repressing ERK activation, if cell contact areas represent the degree of EphrinA-d signaling as they do in the case of FGF9/16/20 signaling. Thus, inductive FGF signaling and inhibitory EphrinA signaling likely accentuate differences in the strength of ERK activation in animal cells.

In *Wnt-NAe* morphants, *Otx* and *Nodal* were expressed in both of the b5.3 daughter cells (b6.5 and b6.6) (Figure 3I and 3J), whereas overexpression of *Wnt-NAe* did not affect *Otx* expression (Figure 3K). This ectopic expression was likely due to the abnormal position of the b6.5 and b6.6 sister cells. In normal embryos, the b6.5 and b6.6 cells were both found in the periphery of the animal hemisphere (Figure 3L), while in the morphants one of them was located at a more interior position (Figure 3M). The boundary between these sister cells is significantly more oblique in the morphants. Because the positions of the rest of the blastomeres of the morphant embryos did not appear to be altered, we could identify these two cells as the daughter cells of b5.3. The mispositioning of the daughter cells of b5.3 likely changed the balance between FGF and EphrinA signaling, because dpERK signal was detected in both of the daughter cells of b5.3 in *Wnt-NAe* morphants (Figure 3N). Thus, this Wnt signaling was not directly involved in transcriptional regulation of *Otx* and *Nodal*.

**Figure 4:**
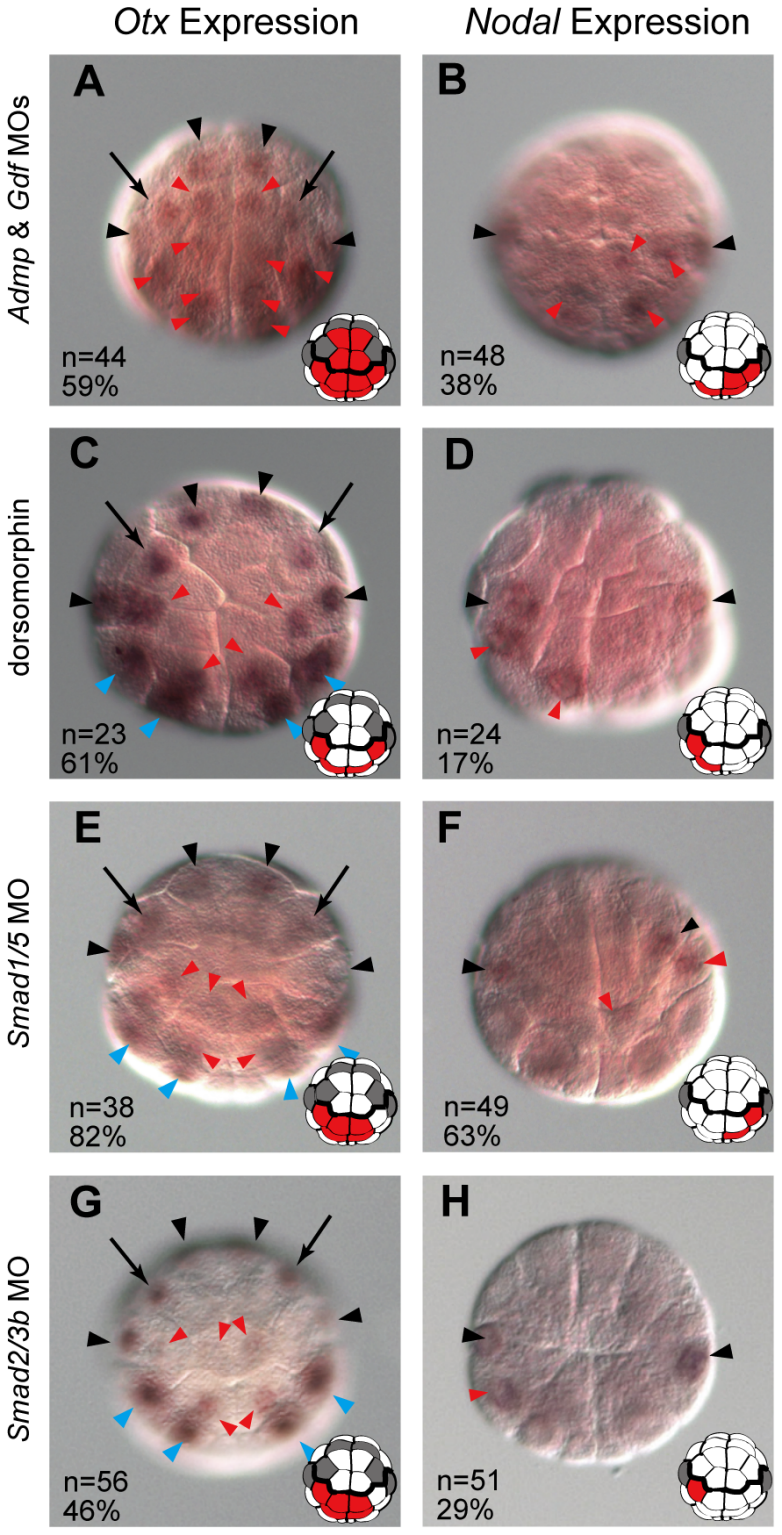
Admp and Gdf1/3-like signaling cooperatively regulates neural fate. Expression of (A, C, E, G) *Otx* and (B, D, F, H) *Nodal* in 32-cell embryos injected with MOs for (A, B) *Admp* and *Gdf1/3*, (E, F) *Smad1/5* and (G, H) *Smad2/3b*, and (C, D) in 32-cell embryos treated with dorsomorphin. Ectopic expression is indicated by red arrowheads. Expression in a6.5 and b6.5 is indicated by black arrowheads, and cryptic expression in a6.7 is indicated by black arrows. Expression in vegetal cells is indicated by blue arrowheads. All embryos are shown in an animal view. The expression of *Otx* and *Nodal* in each panel is depicted. Cells with ectopic expression are colored by red. The expression in a6.5, a6.7 and b6.5 is indicated by dark gray. The scale bar in (A) represents 100 µm.

In *Admp* or *Gdf1/3*-*like* morphants, the expression of *Otx* and *Nodal* was normal (Figure S4; Tables S1 and S2). Since these two molecules are both members of the TGFβ superfamily and might therefore work together, we knocked down these two genes simultaneously. In *Admp* and *Gdf1/3-like* double morphants (*Admp*/*Gdf* morphants hereafter), *Otx* and *Nodal* were ectopically expressed (Figure 4A and 4B; Tables S1 and S2), suggesting redundancy between *Admp* and *Gdf1/3-like*.

Admp signaling is transmitted through the BMP pathway, while GDF1 and GDF3 act through the Activin pathway [24]. A pharmacological inhibitor of BMP signaling, dorsomorphin, resulted in ectopic expression of *Otx* and *Nodal* (Figure 4C and 4D), but an inhibitor of Activin signaling, SB431542, did not (n = 70, 99% for *Otx*; n = 77, 99% for *Nodal*). Knockdown of *Smad1/5*, which encodes an effector transcription factor of the BMP pathway, resulted in ectopic expression of *Otx* and *Nodal* (Figure 4E and 4F). Knockdown of *Smad2/3b*, which encodes an effector of the Activin pathway, also resulted in ectopic expression of *Otx* and *Nodal* (Figure 4G and 4H), although the effect was weaker. These data indicate that the BMP and Activin pathways suppress *Otx* and *Nodal* expression, although the BMP signaling may contribute to this suppression more than Activin signaling.

### Even weak ERK activation induces *Otx* and *Nodal* expression in the absence of Admp/Gdf signaling

**Figure 5:**
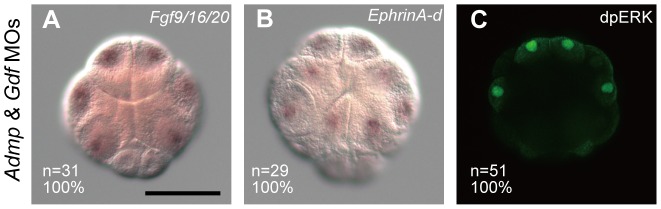
Admp and Gdf1/3-like do not affect FGF/ERK signaling. Expression of (A) *Fgf9/16/20* and (B) *EphrinA-d* in *Admp/Gdf* morphants at the 16-cell stage. (C) Immunostaining of dpERK in an *Admp/Gdf* morphant at the 32-cell stage. The embryo in (A) is shown in a vegetal view and the embryos in (B) and (C) are shown in an animal view. The scale bar in (A) represents 100 µm.

The ectopic expression of *Otx* and *Nodal* in *Admp*/*Gdf* morphants was not due to elevated FGF/ERK signaling, as indicated by the facts that expression of *Fgf9/16/20* and *EphrinA-d* was unaffected at the 16-cell stage (Figure 5A and 5B), and that no ectopic ERK activation was observed in *Admp*/*Gdf* morphants at the 32-cell stage (Figure 5C).

**Figure 6:**
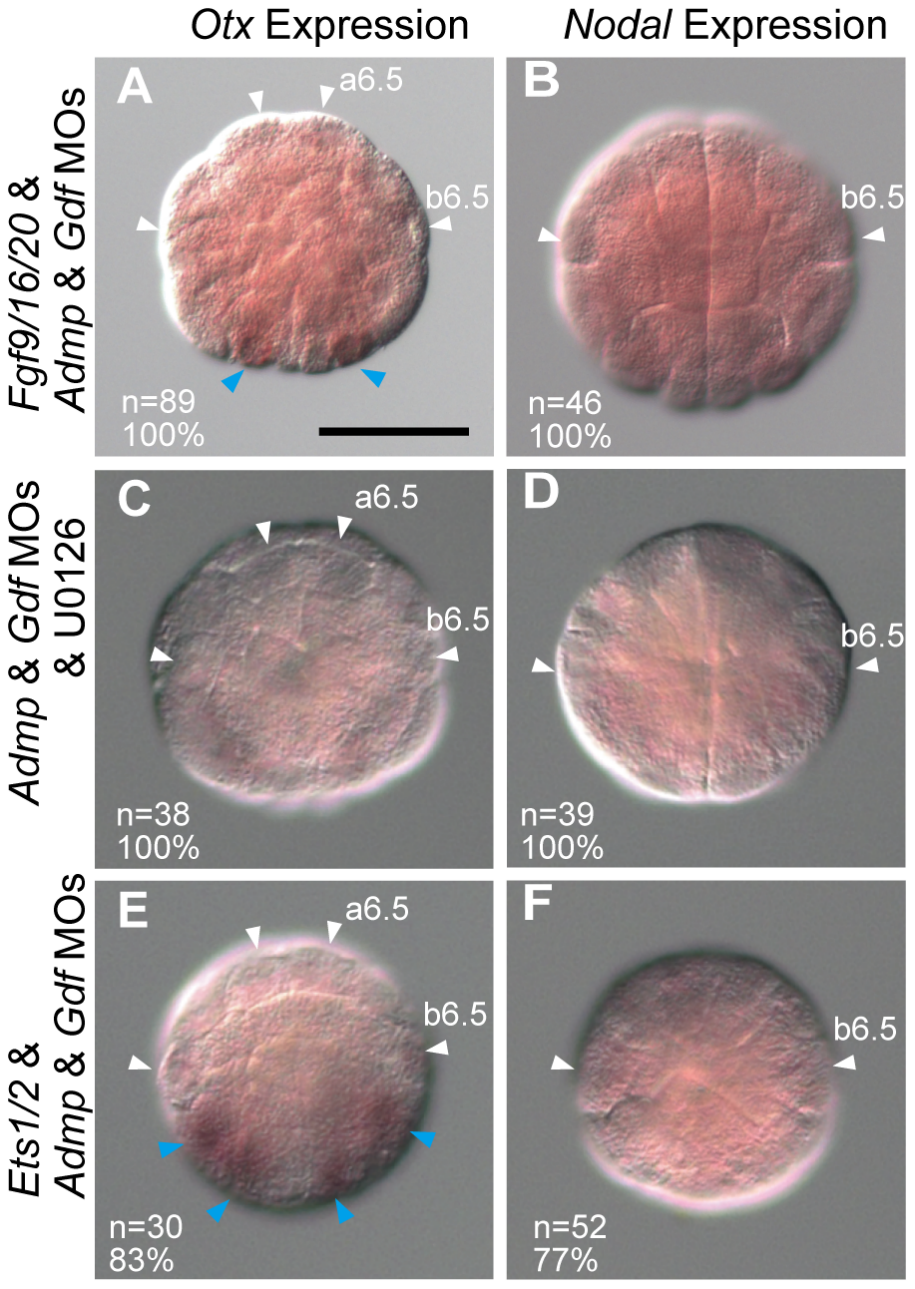
Interplay between FGF/EphrinA signaling and Admp/Gdf signaling. Expression of (A, C, E) *Otx* and (B, D, F) *Nodal* in (A, B) triple *Fgf9/16/20*/*Admp/Gdf* morphants, (C, D) *Admp/Gdf* morphants treated with an MEK inhibitor (U0126), and (E, F) triple *Ets1/2*/*Admp/Gdf* morphants at the 32-cell stage. Loss of expression is indicated by white arrowheads. Expression in vegetal cells is indicated by blue arrowheads. All embryos are shown in an animal view.

Nevertheless, FGF signaling was required for the ectopic expression of *Otx* and *Nodal* in *Admp*/*Gdf* morphants, because *Otx* and *Nodal* were not expressed in triple *Fgf9/16/20*/*Admp*/*Gdf* morphants (Figure 6A and 6B; Tables S1 and S2), *Admp*/*Gdf* morphants treated with an MEK inhibitor U0126 (Figure 6C and 6D), or triple *Ets1/2*/*Admp*/*Gdf* morphants (Figure 6E and 6F). These data suggest that even weak ERK activation that cannot be detected experimentally activates *Otx* and *Nodal* expression in non-neural ectodermal cells, if Admp/Gdf signaling is absent. However, this suppressing activity of Admp/Gdf signaling is limited and the distributions of these signaling molecules are probably unimportant, because overexpression of *Admp* and/or *Gdf1/3-like* rarely suppresses the endogenous expression of *Otx* (Figure S5).

As shown in Table S1, the ectopic expression of *Otx* was observed more frequently in a6.6 than in a6.8, and ectopic expression in a6.6 was observed in all embryos that ectopically expressed *Otx* in a6.8. In addition, expression in a6.7 was also observed in all embryos that ectopically expressed *Otx* in a6.6 and a6.8. Similarly, ectopic expression of *Otx* and *Nodal* in b6.7 was observed in all embryos that expressed these genes in b6.8 (Tables S1 and S2). The expression in b6.6 was observed in all embryos that expressed them in b6.7 and b6.8. These hierarchies within the a- and b-lines (a6.5<a6.7<a6.6<a6.8, b6.5<b6.6<b6.7<b6.8) closely accord with the order of the estimated strength of the EphrinA-d activity (a6.5<a6.7<a6.6<a6.8, b6.5<b6.7<b6.6<b6.8; Figure S3). The only exception is b6.6 and b6.7, and notably the contact area with FGF-expressing vegetal cells is estimated to be larger in b6.6 than in b6.7 [12]. Therefore, the above observation supports the estimation of EphrinA-d signaling strength by the 3D-virtual embryos.

**Figure 7:**
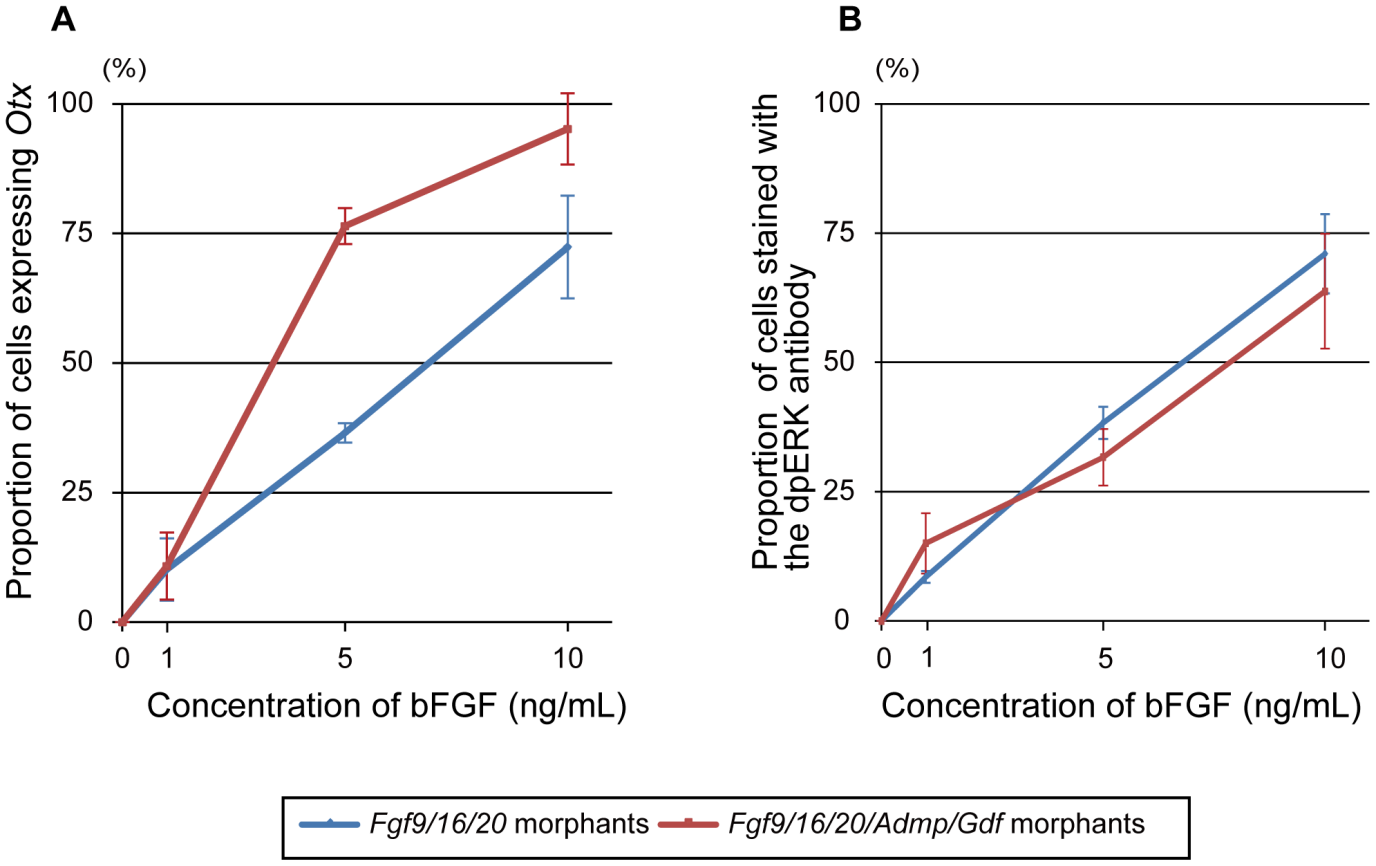
*Fgf9/16/20/Admp/Gdf1/3-like* morphants respond more sensitively to human bFGF than *Fgf9/16/20* morphants. Proportion of (A) cells expressing *Otx* and (B) cells stained with the dpERK antibody to all of the animal cells at concentrations of 0, 1, 5 and 10 ng/mL of human bFGF. Blue and red lines indicate proportion of *Fgf9/16/20* morphants and *Fgf9/16/20/Admp/Gdf1/3-like* morphants. Error bars in (A) and (B) indicate standard errors between two and three independent experiments, respectively.

Our data suggested that *Admp/Gdf* morphants are more sensitive to FGF signaling than normal embryos. Indeed, we found that *Fgf9/16/20*/*Admp*/*Gdf* morphants responded more sensitively to human bFGF added to the sea water than *Fgf9/16/20* morphants; namely, *Fgf9/16/20*/*Admp*/*Gdf* morphants expressed *Otx* more frequently with increasing concentrations of bFGF (Figure 7A). On the other hand, there was no significant difference in the proportion of cells stained with the dpERK antibody (Figure 7B). At an intermediate concentration (5 ng/mL), 76% of the animal cells in *Fgf9/16/20*/*Admp*/*Gdf* morphants and 37% in *Fgf9/16/20* morphants expressed *Otx* (Figure 7A), while dpERK signal was detected in 31% and 38% of cells in these morphants (Figure 7B). Thus, weak FGF signaling that is experimentally undetectable by dpERK immunostaining can activate *Otx* expression, and this weak signaling is inhibited by Admp/Gdf signaling. At the same time, the dose-dependent response of *Otx* activation indicates that differential FGF/ERK signaling strength alone cannot explain the threshold response.

### Admp/Gdf signaling directly suppresses the action of FGF-responsive enhancer to evoke a robust threshold reaction

**Figure 8:**
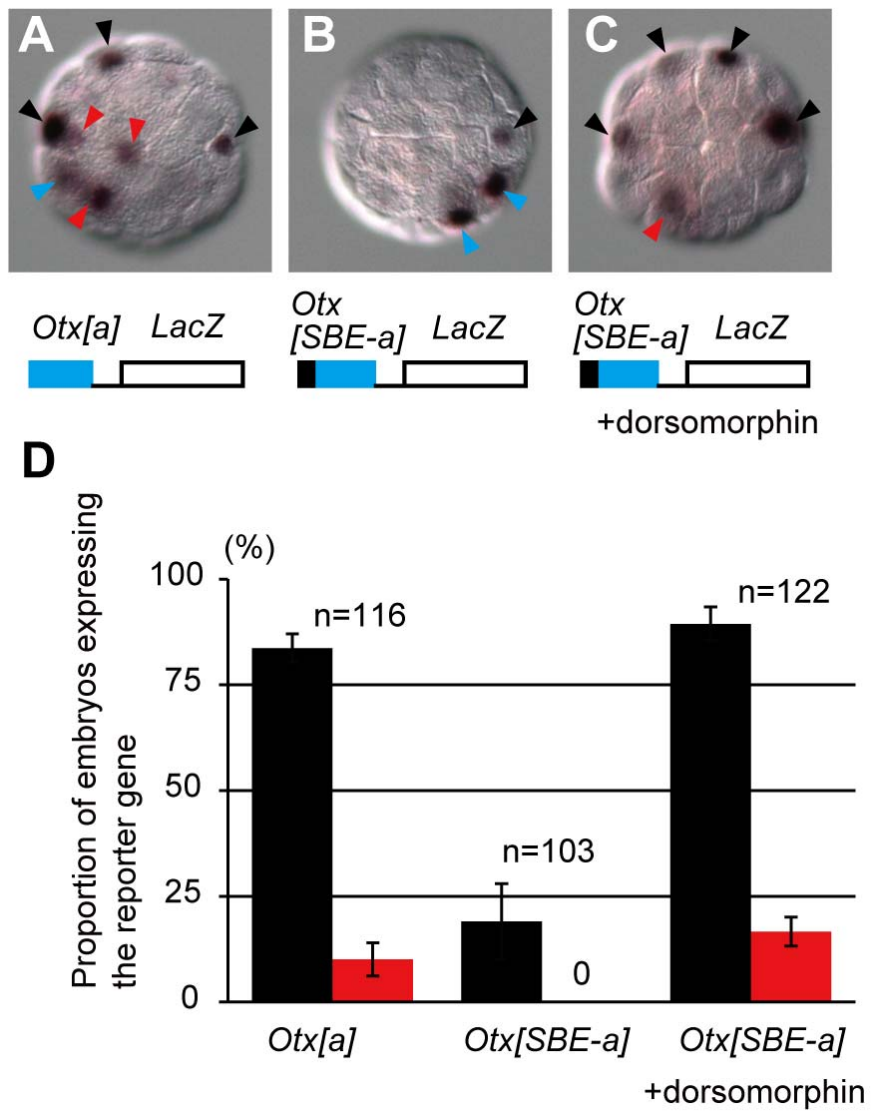
SBEs suppress the activity of FGF-responsive elements within the *Otx* a-enhancer. (A–C) In situ hybridization showing a *LacZ* reporter gene expression in embryos electroporated with (A) *Otx[a]>LacZ* and (B, C) *Otx[SBE-a]>LacZ*. The embryo shown in (C) was treated with dorsomorphin. Black arrowheads indicate reporter gene expression in cells with endogenous *Otx* expression. Red and blue arrowheads indicate ectopic expression and expression in vegetal cells, respectively. (D) Proportion of embryos expressing the reporter gene in a6.5 and b6.5 (black bars) and in the epidermal lineage (red bars). Error bars indicate standard errors between three independent experiments. Note that due to mosaic incorporation of the fusion gene, not all ectodermal cells have the transgene.

Previous studies [16], [25] showed that an upstream region (a-element) of *Otx* is responsible for *Otx* expression in a6.5 blastomeres at the 32-cell stage. GATA and Ets transcription factors activated by the ERK signaling pathway bind to the a-element [16] (Figure S6A). Thus, we used a previously characterized reporter construct, in which the a-element and the minimal promoter region of the *Brachyury* gene were fused to the *LacZ* coding sequence [16] (*Otx[a]>LacZ*). This reporter construct was electroporated into fertilized eggs, and expression of *LacZ* mRNA was examined at the 32-cell stage. In addition to strong signal in a6.5 and b6.5 [16], we found weak signals in non-neural ectodermal cells in 10% of the experimental embryos (Figure 8A and 8D).

By examining the genomic sequence around the a-element of *Otx*, we identified two putative Smad-binding elements and one binding element for Smad4, a co-factor of regulatory Smad proteins [26], [27], within the 100-bp upstream region of the a-element (Figure S6A). When the region containing these Smad-binding elements (collectively called SBEs) was placed upstream of the a-element (*Otx[SBE-a]>LacZ*), *LacZ* was expressed specifically in the neural lineage, although the number of embryos expressing the reporter was reduced (Figure 8B and 8D). Treatment with dorsomorphin again induced ectopic expression of *LacZ* and enhanced overall expression, indicating that the SBEs work downstream of BMP signaling to weaken the activity of the enhancer (Figure 8C and 8D).

A *Nodal* cis-regulatory element responsible for expression in the neural lineage of cells (*Nodal*-a-element) was also identified previously [17] (Figure S6B).The *Nodal*-a-element induced the reporter gene expression in the anterior and posterior animal cells (*Nodal[a]>LacZ*), probably because it lacks FoxA-a binding sites. As in the case of *Otx*, this *Nodal*-a-element also induced non-neural expression (Figure S7A). We found one regulatory Smad binding site and one Smad4 binding site downstream of this enhancer. These SBEs suppressed *LacZ* reporter expression, when connected to the *Nodal*-a-element, and this suppression was abolished by dorsomorphin treatment (Figure S7B–D). Thus, Admp/Gdf signaling directly suppresses the activity of *Otx* and *Nodal* enhancers to evoke a robust threshold reaction.

## Discussion

Previous studies showed that differential FGF signaling from vegetal cells to animal cells plays a primary role in specific expression of *Otx* and *Nodal* [9], [12], [16], [17], [19]. However, it was unclear why non-neural ectodermal cells, which still receive FGF signals but at lower levels, fail to activate *Otx* and *Nodal* at all. Here, we showed that EphrinA-d, which antagonizes FGF signaling [20]–[22], amplifies the difference in ERK activity between ectodermal cells, as shown by dpERK immunostaining. Even below the detection limit, weak ERK activation occasionally activates *Otx* and *Nodal* expression, probably due to the inherently stochastic nature of signaling pathways and transcriptional activation [8]. The activity of *Otx* and *Nodal* transcriptional enhancers is weakened by Admp/Gdf signaling through the SBEs within the enhancers. The silencing activity of the SBEs is relatively weak and never overcomes fully activated enhancing activity. Thus, cooperation of multiple signaling pathways evokes a robust threshold reaction.

However, this cooperation cannot perfectly evoke a threshold response, because some embryos express *Otx* in a6.7 (6% in a previous assay [12] and 35% in our assay). As previously shown [12], FGF signaling is expected to be stronger in a6.7 than in a6.6 and a6.8. EphrinA-d signaling is expected to be stronger in a6.7 than in a6.5, and weaker in a6.7 than in a6.6 and a6.8, if cell contact areas with EphrinA-d-expressing cells simply represent the degree of EphrinA-d signaling. It is very likely that the sum of the positive and negative signaling activities in a6.7 is near the threshold, and consequently a6.7 occasionally activates *Otx*.

*Admp* is expressed in the posterior vegetal cells, and *Gdf1/3-like* is expressed in all of the cells in the animal hemisphere. Although these two factors are probably two major factors activating the BMP and Activin pathways, several members of the TGFβ-superfamily, including *BMP2/4* and *BMP3* are also expressed maternally [10]. In addition, TGFβ-superfamily molecules must be processed to become functional. Therefore, it is difficult to measure how these signaling molecules are distributed. However, because Admp and Gdf1/3-like cannot repress endogenous *Otx* and *Nodal* expression when overexpressed, the distributions of these two signaling molecules are probably unimportant for controlling *Otx* and *Nodal* expression.

Intriguingly, we found that knockdown of either of *Smad1/5* or *Smad2/3b* causes ectopic expression of *Otx* and *Nodal*, while knockdown of either *Admp* or *Gdf1/3-like* does not produce an obvious phenotype. There are several possible explanations for this observation. *Admp* and *Gdf1/3-like* might not be fully knocked-down by the MOs we used, or other maternally expressed TGFβ-superfamily members might function redundantly. Additionally, there might be crosstalk between the BMP-signaling and Activin signaling pathways [28]. Nonetheless, the role of BMP/Activin-signaling we demonstrated in the regulation of *Otx* and *Nodal* expression is clear.

Transcriptional repressors play an important role in delineating sharp boundaries of gene expression [29]. For example, in *Drosophila* embryos, repressors that antagonize Bicoid activity are responsible for converting gradients into threshold responses [4]. Although reverse gradients can make a steep gradient, transcriptional repressors are often also required to repress residual activities, as in the case of neural cells of the *Ciona* embryo. A similar mechanism might function in a variety of developmental processes in which multiple signaling pathways are involved.

Similar to neural fate specification in the *Ciona* embryo, neural induction in *Xenopus* embryos involves BMP and FGF signaling. According to the most widely accepted “default model”, BMP inhibition is both necessary and sufficient for neural induction of vertebrate embryos [30], while FGF has an instructive role [31]–[34]. In addition, FGF signaling inhibits BMP signaling by phosphorylating Smad1, leading to the degradation of Smad1 [35], [36]. Inhibition of BMP signaling also induces FGF expression [33]. These mechanisms do not seem to be the principal mechanism of neural induction in *Ciona* embryos. However, it would be interesting to investigate whether the mechanism we describe here in *Ciona* embryos also functions in the vertebrate organizers.

Although it is not involved in evoking a threshold reaction, Wnt signaling is required for the proper spatial expression of *Otx* and *Nodal*. Our finding that Admp, Gdf1/3-like, and Wnt signaling regulate *Otx* and *Nodal* expression in the neural lineage is based on an unbiased and systematic analysis of signaling molecule genes expressed at the 16-cell stage in *Ciona* embryos. Because Admp/Gdf signaling and Wnt signaling do not play an instructive role in *Otx* and *Nodal* expression in the neural lineage, their involvement might have been difficult to uncover apart from such a comprehensive and unbiased approach.

## Materials and Methods

### Ascidian embryos and pharmacological inhibitors

*C. intestinalis* adults were obtained from the National Bio-Resource Project for *Ciona*. cDNA clones were obtained from our EST clone collection [37]. Inhibitors of BMP signaling (dorsomorphin; Wako), Activin signaling (SB431542, Sigma), and MEK signaling (U0126, Promega) were used at concentrations of 100 µM, 5 µM, and 10 µM, respectively. To examine responses to FGF, we used human recombinant bFGF (Sigma). SB431542 and U0126 were shown to work properly in the *Ciona* embryo in previous studies [11], [19]. As shown in Figure S8A, dorsomorphin treatment inhibits phosphorylation of Smad1/5; Western blotting with polyclonal antibodies against phosphorylated Smad5 (Abcam, ab92698) showed that treatment with human BMP4 (100 ng/mL; humanzyme) evoked hyper-phosphorylation of Smad1/5 in the 32-cell embryo and dorsomorphin (50 µM) inhibited this phosphorylation. After stripping the membrane, we performed Western blotting with antibodies for β-tubulin for a loading control (Sigma, T5293).

### Gene knockdown, overexpression and whole-mount in situ hybridization

The morpholino oligonucleotides (MOs) (Gene Tools, LLC) for *FoxA-a*, *Admp*, *Gdf1/3-like*, *Fgf9/16/20*, *Wnt-NAe*, *EphrinA-d* and Ets1/2 were the same ones that we used in a previous study [13]. We designed an additional MO for *Wnt-NAe* (5′-TGTAAATGAAGACAACAGTTTAGAG-3′), which produced the same phenotype (ectopic *Otx* expression) as the original one, so only the results obtained with the second MO are shown. We also designed MOs for *Smad1/5* (5′-AACAACTTCTCCACACAACAACCTG-3′) and *Smad2/3b* (5′-CATATTTACTCTCAATGTTCGATGT-3′) in the present study. All of these MOs were designed for blocking translation. The specificity of the *Smad1/5* MO was confirmed by Western blotting. As described above, in embryos treated with human BMP4, phosphorylated Smad1/5 was detectable. When embryos injected with the *Smad1/5* MO were treated with human BMP4, phosphorylated Smad1/5 was rarely detected (Figure S8B). The specificity of the *Smad2/3b* MO was confirmed by a rescue experiment: when we injected the *Smad2/3b* MO with a synthetic *Smad2/3b* mRNA that the MO cannot bind, ectopic expression of *Otx*, which is a phenotype of *Smad2/3b* morphants, was not observed (Figure S8C).

Synthetic overexpression transcripts were prepared from cDNA cloned into pBluescript RN3 vector [38] by in vitro transcription using a commercially available kit (mMESSAGE mMACHINE T3, Ambion), and injected into fertilized eggs at a concentration of 1 mg/mL. DIG-RNA probes for whole-mount in situ hybridization were synthesized by in vitro transcription with T7 RNA polymerase. The detailed procedure has been described previously [10].

### Immunostaining

To detect activation of the receptor-tyrosine kinase cascade, embryos were fixed with 3.7% formaldehyde and were treated with 3% H_2_O_2_ for 30 minutes to quench endogenous peroxidase activity, and then incubated overnight with mouse anti-dpERK (1∶1000, Sigma, M9692) in Can-Get-Signal-Immunostain Solution B (TOYOBO). The signal was visualized with a TSA Kit (Invitrogen) using HRP-conjugated goat anti-mouse IgG and Alexa Fluor 488 tyramide. To visualize cell morphology, F-actin was stained with Alexa Fluor 555–conjugated Phalloidin (Invitrogen).

### Contact area of cells with surrounding cells expressing *EphrinA-d* and *Fgf9/16/20*

The contact surfaces of individual animal blastomeres of the 32-cell embryo with cells expressing *EphrinA-d* were calculated using the 3D-virtual embryo [12]. Given the delay between gene expression and protein translation, we assumed that cells descended from *EphrinA-d*-expressing cells at the 16-cell stage express EphrinA-d protein at the 32-cell stage. Because EphrinA-d is GPI-anchored, we ruled out autoregulatory effects. The contact surfaces of individual animal blastomeres of the 32-cell embryo with anterior vegetal cells expressing *Fgf9/16/20* were previously calculated [12]. However, because *Fgf9/16/20* is also expressed in posterior vegetal cells, we included the posterior vegetal cells in our present calculations using the 3D-virtual embryo [12].

### Analysis of cis-regulatory elements

DNA constructs for examining regulatory elements were introduced by electroporation [39]. Cis-regulatory elements of *Otx* and *Nodal* were fused to the *Brachyury* and *Fog* basal promoters [17], [40], [41], respectively. *LacZ* was used as a reporter gene. The expression of *LacZ* was examined by in situ hybridization.

## Supporting Information

Figure S1 Expression of genes analyzed in the present study. (A, B) Expression of *Otx* and *Nodal* in the animal hemisphere at the 32-cell stage. (C–H) Expression of *FoxA-a*, *Fgf9/16/20*, *Wnt-NAe*, *EphrinA-d*, *Admp*, and *Gdf1/3-like* expression at the 16-cell stage. Blastomeres that express the genes indicated are shown in gray with cell identities. The illustration is based on a previous study [1].
(TIF)

Figure S2 Expression of *Otx* in 32-cell embryos injected with synthetic *Fgf9/16/20* RNA. (A) Expression of *Otx* in embryos that were developed from eggs injected with the synthetic mRNA. *Otx* was expressed in the entire animal hemisphere. (B) We injected the synthetic mRNA into one posterior animal cell of the 8-cell embryos. Strong signal was detected in the entire cytoplasm and nuclei of the four descendants of the injected blastomere (enclosed by a dotted cyan line) and the neighbors (red arrowheads). All embryos are shown in an animal view.
(TIF)

Figure S3 Estimated contact surfaces of individual animal blastomeres of the 32-cell embryo with cells expressing *EphrinA-d* and *Fgf9/16/20*. Using the 3D-virtual embryo tool developed in a previous study [2], contact surfaces were estimated. We considered autocrine signaling only for Fgf9/16/20, because EphrinA-d is a GPI-anchored protein. The contact surfaces with *Fgf9/16/20*-expressing cells are basically the same data as in the previous study [2]. However, because the previous study did not consider posterior vegetal cells expressing *Fgf9/16/20*, we recalculated the contact surfaces with all of the vegetal cells expressing *Fgf9/16/20* using the 3D-virtual embryo tool.
(TIF)

Figure S4 Expression of *Otx* and *Nodal* is not affected in *Admp* or *Gdf1/3-like* morphants. Expression of (A, C) *Otx* and (B, D) *Nodal* in 32-cell embryos injected with MOs for (A, B) *Admp*, and (C, D) *Gdf1/3-like*. Expression in a6.5 and b6.5 is indicated by black arrowheads, and expression in a6.7 is indicated by an arrow. Expression of *Otx* in vegetal cells is indicated by blue arrowheads. All embryos are shown in an animal view.
(TIF)

Figure S5 Overexpression of *Admp* and/or *Gdf1/3-like* rarely affects *Otx* expression. Expression of *Otx* in 32-cell embryos injected with RNAs of (A) *Admp*, (B) *Gdf1/3-like* and (C) *Admp* and *Gdf1/3-like*. Expression in a6.5 and b6.5 is indicated by black arrowheads, and expression in a6.7 is indicated by an arrow. Expression of *Otx* in vegetal cells is indicated by blue arrowheads. All embryos are shown in an animal view.
(TIF)

Figure S6 Putative SMAD binding elements (SBEs) and the a-elements in the (A) *Otx* and (B) *Nodal* upstream sequences. The a-elements are underlined. GATA-a binding sites and Ets binding sites are shown in light blue and green, respectively [3]. SBEs are shown in red. Sequences connected to the a-elements in *Otx[SBE-a]>LacZ* and *Nodal[a-SBE]>LacZ* are enclosed by boxes. The scaffold numbers and genomic positions of these sequences are shown in both ends.
(TIF)

Figure S7 SBEs suppress the activity of FGF-responsive elements within the *Nodal* a-enhancer. Expression of a *LacZ* reporter gene in embryos electroporated with (A) *Nodal[a]>LacZ* and (B, C) *Nodal[a-SBE]>LacZ*, as is revealed by in situ hybridization. The embryo shown in (C) was treated with dorsomorphin. Black arrowheads indicate reporter gene expression in b6.5. Red arrowheads indicate ectopic expression. (D) Proportion of embryos expressing the reporter gene in a6.5 and b6.5 (black bars) and in the epidermal lineage (red bars). Error bars indicate standard error between three independent experiments.
(TIF)

Figure S8 Specificity of dorsomorphin and the MOs for *Smad1/5* and *Smad2/3b*. (A) Western blotting showing specificity of dorsomorphin. In *Ciona* embryo treated with human BMP4, phosphorylated Smad1/5 was detected with anti-phosphorylated Smad5 antibodies. Phosphorylated Smad1/5 was not detected in embryos treated with dorsomorphin. (B) Western blotting showing specificity of the *Smad1/5* MO. Phosphorylated Smad1/5, which was detectable in embryos treated with human BMP4, was hardly detected in embryos injected with the *Smad1/5* MO. β-tubulin was used for loading controls. (C) Expression of *Otx* in embryos injected with the *Smad2/3b* MO and a synthetic mRNA of *Smad2/3b* that the MO cannot bind. Ectopic expression was not seen in 96.7% of embryos examined (n = 30). Black and blue arrowheads indicate expression of *Otx* in the animal and vegetal hemispheres, which is seen in normal embryos.
(TIF)

Table S1 Number of embryos expressing *Otx* in designated blastomeres of control and morphant embryos. ^*1^ Note that *Otx* was not expressed in combinations of cells not shown in this table; ‘+’ indicates the expression of *Otx*. ^*2^ The expression in the a- and b-line blastomeres of the same embryos was counted separately.
(DOCX)

Table S2 Number of embryos expressing *Nodal* in designated blastomeres of control and morphant embryos. ^*1^ Note that *Nodal* was not expressed in combinations of cells not shown in this table; ‘+’ indicates the expression of *Nodal*. ^*2^ The expression in the a- and b-line blastomeres of the same embryos was counted separately.
(DOCX)
