# Supplementary figures and images for: Multiple Signaling Pathways Coordinate to Induce a Threshold Response in a Chordate Embryo
Source: PLoS Genet. 2013 Oct 3;9(10):e1003818. doi: 10.1371/journal.pgen.1003818 (PMC3789818; doi:10.1371/journal.pgen.1003818)

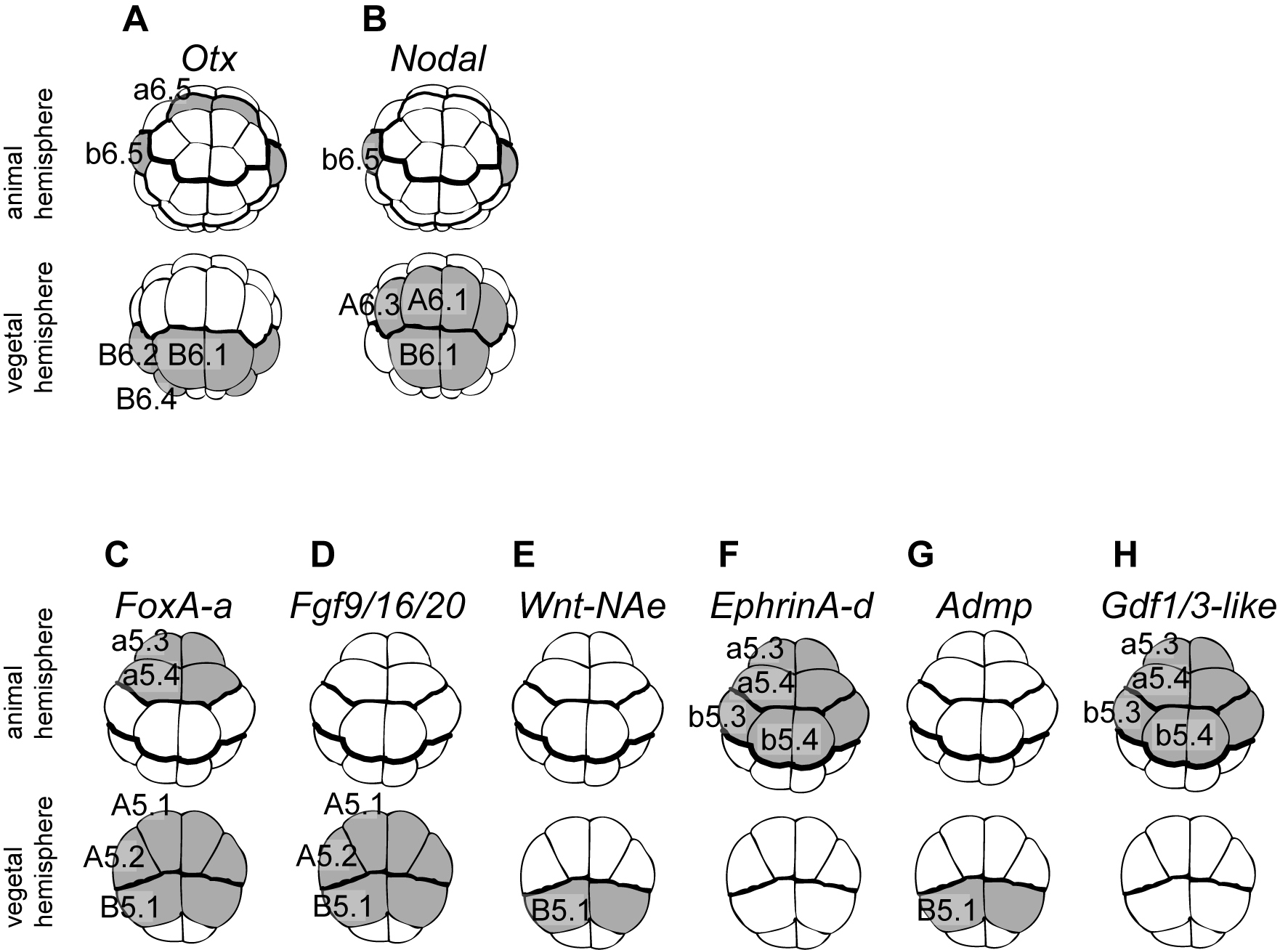

Supplement: Figure S1 — Expression of genes analyzed in the present study. (A, B) Expression of Otx and Nodal in the animal hemisphere at the 32-cell stage. (C–H) Expression of FoxA-a, Fgf9/16/20, Wnt-NAe, EphrinA-d, Admp, and Gdf1/3-like expression at the 16-cell stage. Blastomeres that express the genes indicated are shown in gray with cell identities. The illustration is based on a previous study [1]. (TIF) [file pgen.1003818.s001.tif]

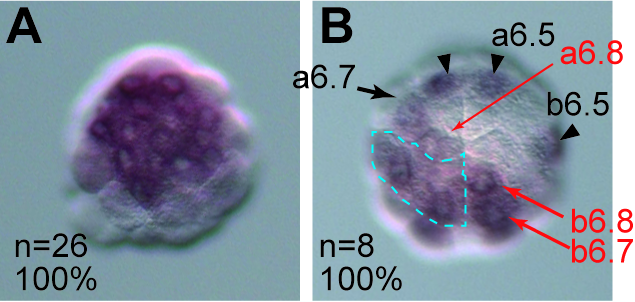

Supplement: Figure S2 — Expression of Otx in 32-cell embryos injected with synthetic Fgf9/16/20 RNA. (A) Expression of Otx in embryos that were developed from eggs injected with the synthetic mRNA. Otx was expressed in the entire animal hemisphere. (B) We injected the synthetic mRNA into one posterior animal cell of the 8-cell embryos. Strong signal was detected in the entire cytoplasm and nuclei of the four descendants of the injected blastomere (enclosed by a dotted cyan line) and the neighbors (red arrowheads). All embryos are shown in an animal view. (TIF) [file pgen.1003818.s002.tif]

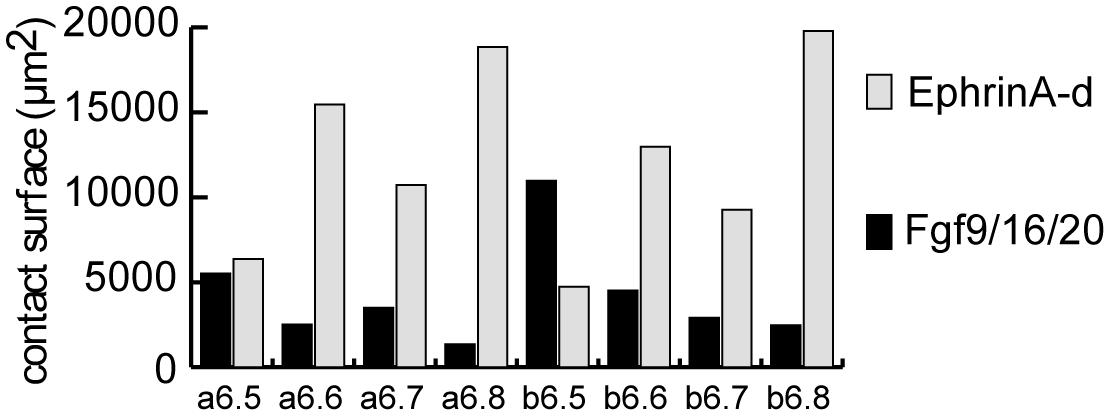

Supplement: Figure S3 — Estimated contact surfaces of individual animal blastomeres of the 32-cell embryo with cells expressing EphrinA-d and Fgf9/16/20. Using the 3D-virtual embryo tool developed in a previous study [2], contact surfaces were estimated. We considered autocrine signaling only for Fgf9/16/20, because EphrinA-d is a GPI-anchored protein. The contact surfaces with Fgf9/16/20-expressing cells are basically the same data as in the previous study [2]. However, because the previous study did not consider posterior vegetal cells expressing Fgf9/16/20, we recalculated the contact surfaces with all of the vegetal cells expressing Fgf9/16/20 using the 3D-virtual embryo tool. (TIF) [file pgen.1003818.s003.tif]

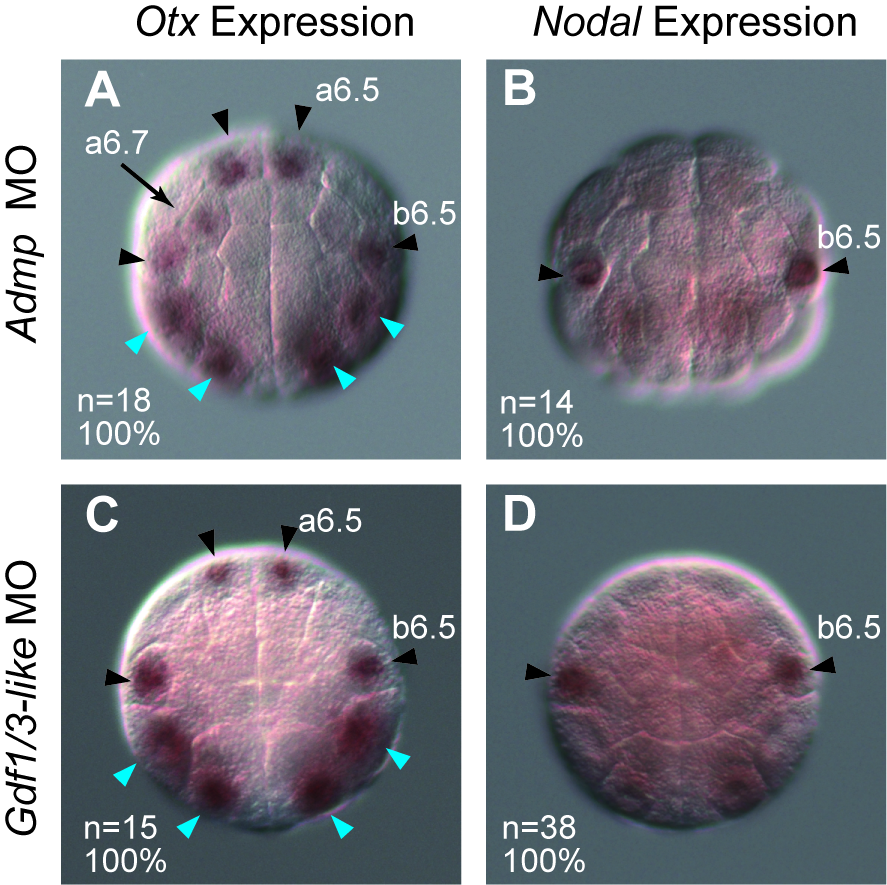

Supplement: Figure S4 — Expression of Otx and Nodal is not affected in Admp or Gdf1/3-like morphants. Expression of (A, C) Otx and (B, D) Nodal in 32-cell embryos injected with MOs for (A, B) Admp, and (C, D) Gdf1/3-like. Expression in a6.5 and b6.5 is indicated by black arrowheads, and expression in a6.7 is indicated by an arrow. Expression of Otx in vegetal cells is indicated by blue arrowheads. All embryos are shown in an animal view. (TIF) [file pgen.1003818.s004.tif]

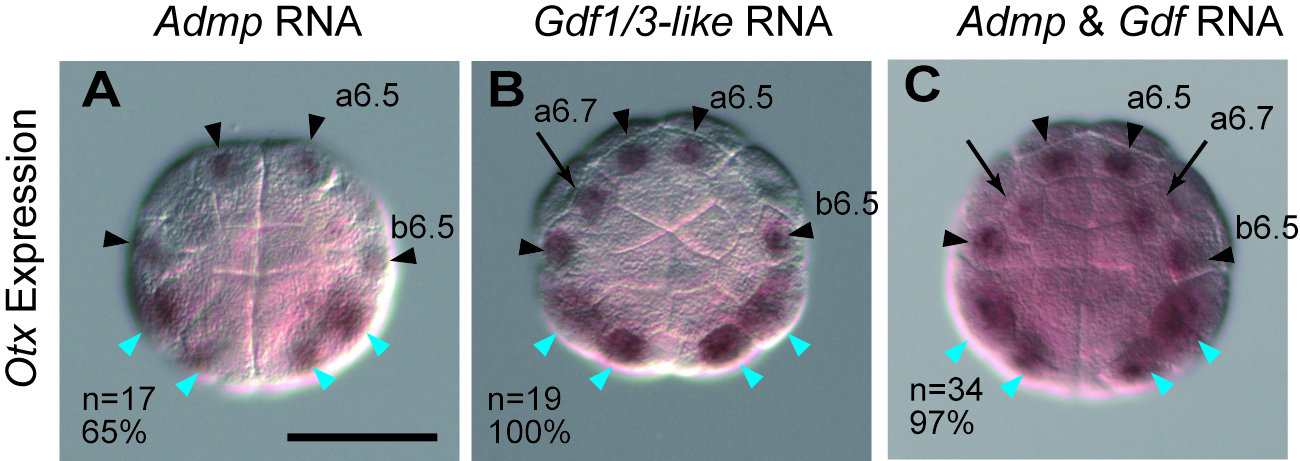

Supplement: Figure S5 — Overexpression of Admp and/or Gdf1/3-like rarely affects Otx expression. Expression of Otx in 32-cell embryos injected with RNAs of (A) Admp, (B) Gdf1/3-like and (C) Admp and Gdf1/3-like. Expression in a6.5 and b6.5 is indicated by black arrowheads, and expression in a6.7 is indicated by an arrow. Expression of Otx in vegetal cells is indicated by blue arrowheads. All embryos are shown in an animal view. (TIF) [file pgen.1003818.s005.tif]

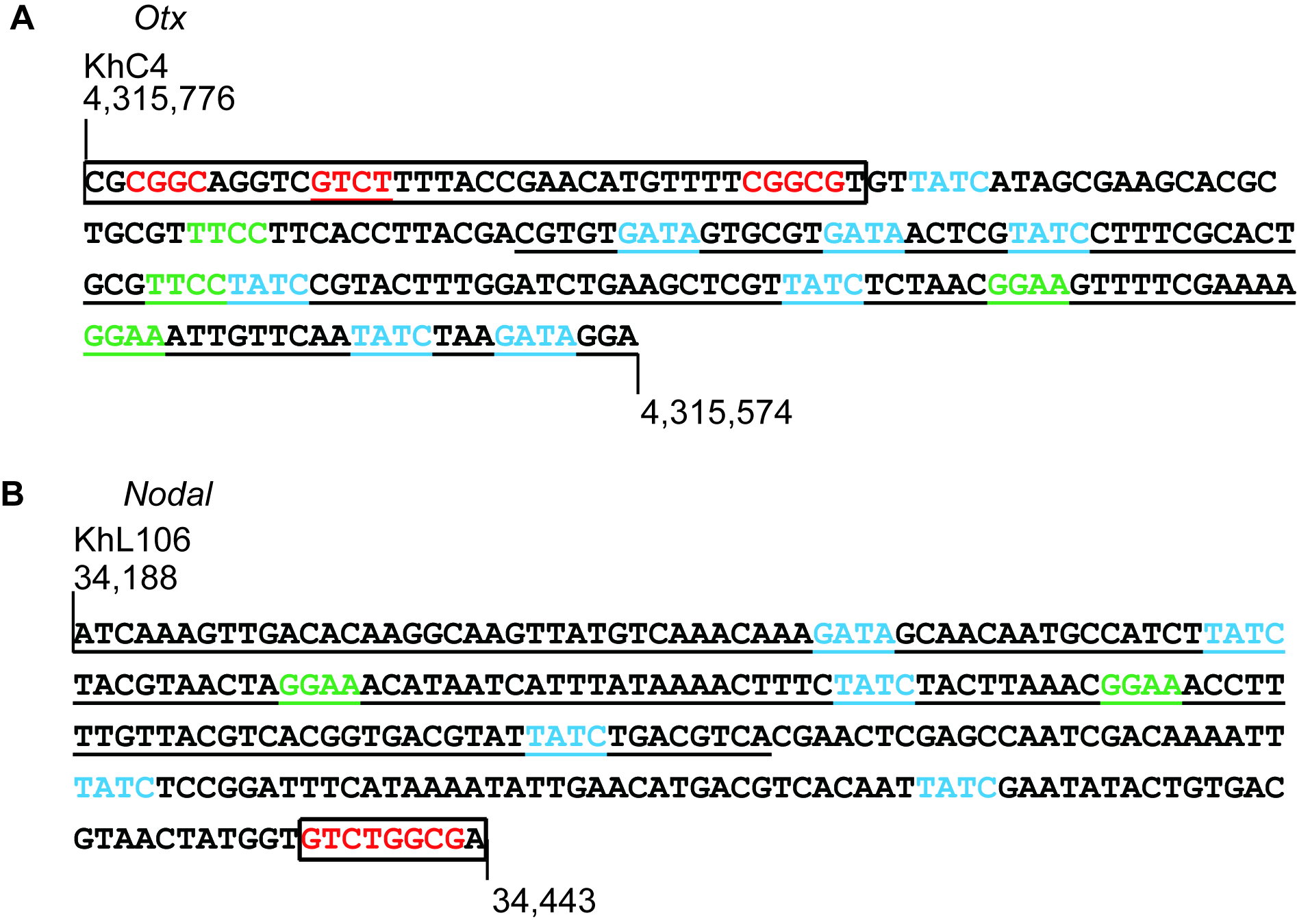

Supplement: Figure S6 — Putative SMAD binding elements (SBEs) and the a-elements in the (A) Otx and (B) Nodal upstream sequences. The a-elements are underlined. GATA-a binding sites and Ets binding sites are shown in light blue and green, respectively [3]. SBEs are shown in red. Sequences connected to the a-elements in Otx[SBE-a]>LacZ and Nodal[a-SBE]>LacZ are enclosed by boxes. The scaffold numbers and genomic positions of these sequences are shown in both ends. (TIF) [file pgen.1003818.s006.tif]

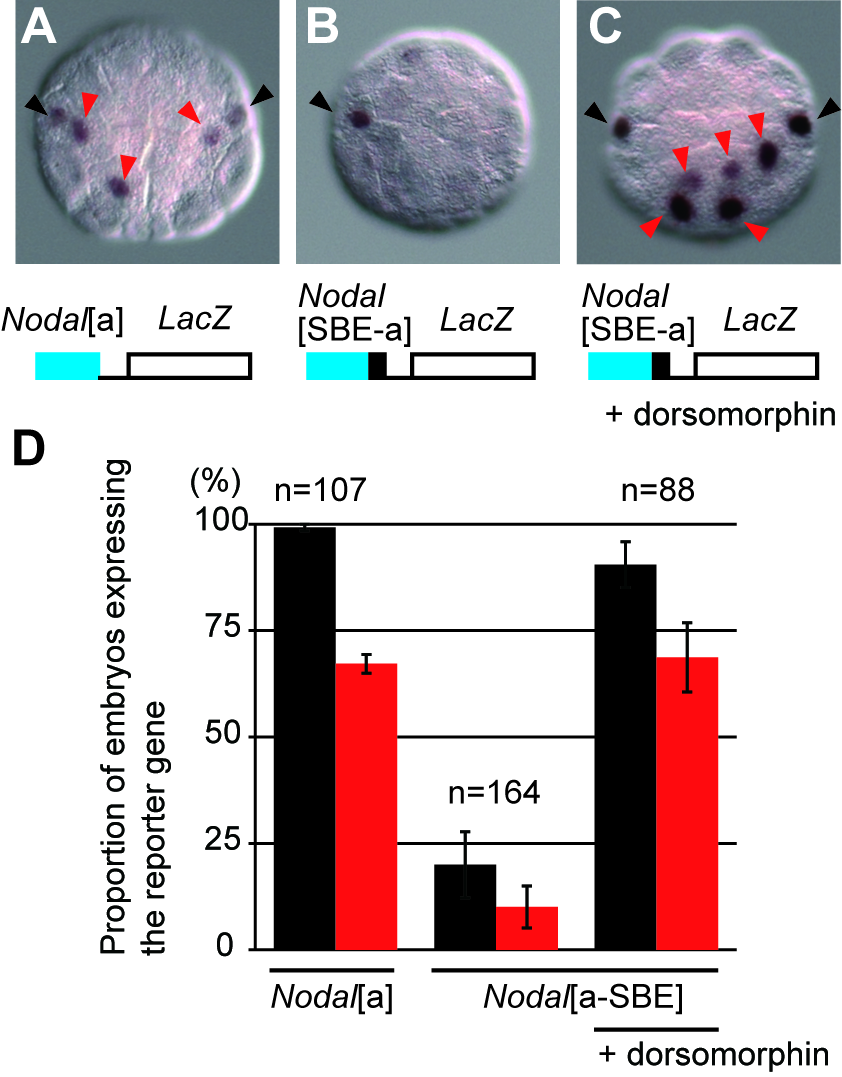

Supplement: Figure S7 — SBEs suppress the activity of FGF-responsive elements within the Nodal a-enhancer. Expression of a LacZ reporter gene in embryos electroporated with (A) Nodal[a]>LacZ and (B, C) Nodal[a-SBE]>LacZ, as is revealed by in situ hybridization. The embryo shown in (C) was treated with dorsomorphin. Black arrowheads indicate reporter gene expression in b6.5. Red arrowheads indicate ectopic expression. (D) Proportion of embryos expressing the reporter gene in a6.5 and b6.5 (black bars) and in the epidermal lineage (red bars). Error bars indicate standard error between three independent experiments. (TIF) [file pgen.1003818.s007.tif]

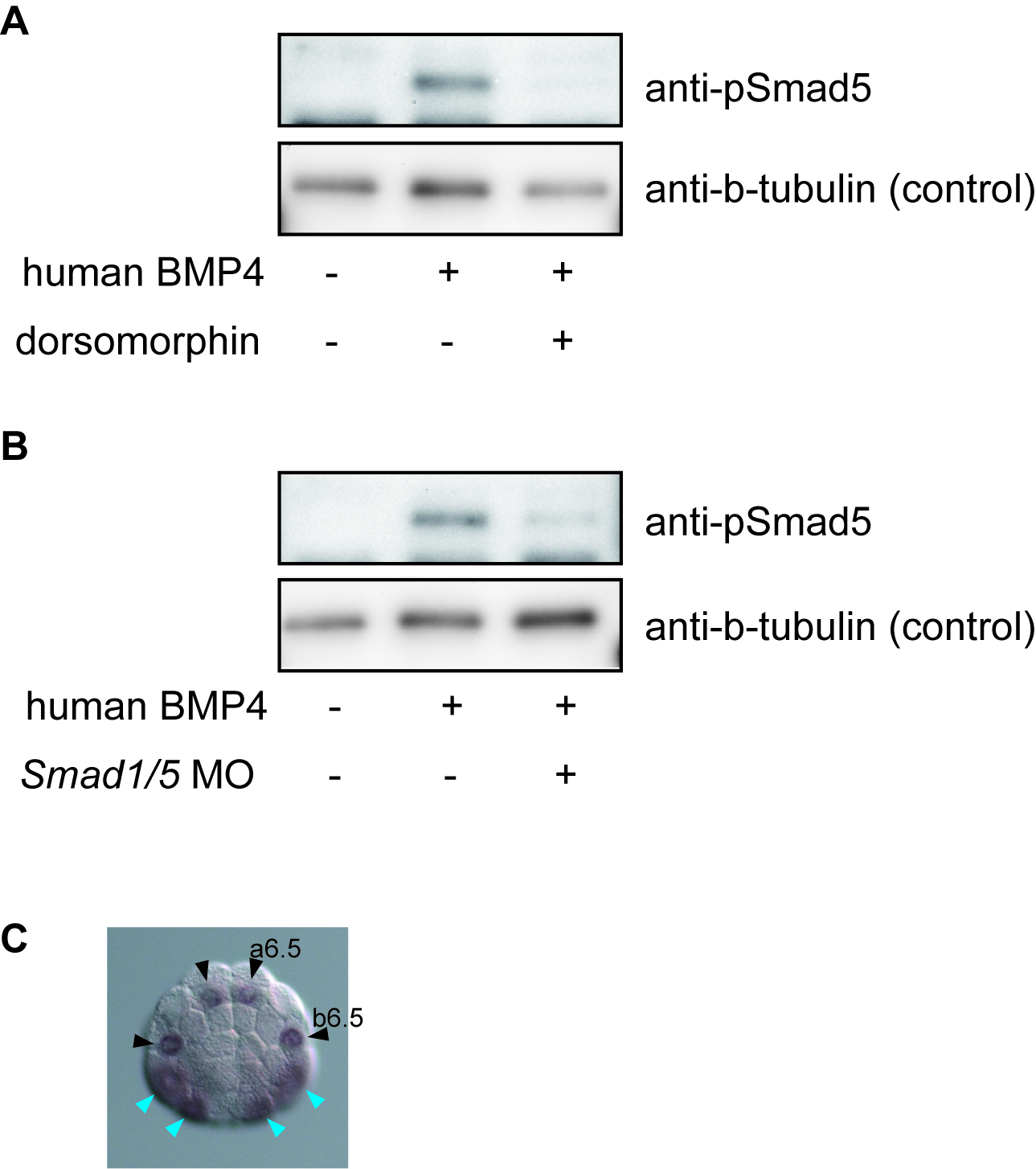

Supplement: Figure S8 — Specificity of dorsomorphin and the MOs for Smad1/5 and Smad2/3b. (A) Western blotting showing specificity of dorsomorphin. In Ciona embryo treated with human BMP4, phosphorylated Smad1/5 was detected with anti-phosphorylated Smad5 antibodies. Phosphorylated Smad1/5 was not detected in embryos treated with dorsomorphin. (B) Western blotting showing specificity of the Smad1/5 MO. Phosphorylated Smad1/5, which was detectable in embryos treated with human BMP4, was hardly detected in embryos injected with the Smad1/5 MO. β-tubulin was used for loading controls. (C) Expression of Otx in embryos injected with the Smad2/3b MO and a synthetic mRNA of Smad2/3b that the MO cannot bind. Ectopic expression was not seen in 96.7% of embryos examined (n = 30). Black and blue arrowheads indicate expression of Otx in the animal and vegetal hemispheres, which is seen in normal embryos. (TIF) [file pgen.1003818.s008.tif]
