# Supplementary material for: Multiple Signaling Pathways Coordinate to Induce a Threshold Response in a Chordate Embryo
Source: PLoS Genet. 2013 Oct 3;9(10):e1003818. doi: 10.1371/journal.pgen.1003818 (PMC3789818; doi:10.1371/journal.pgen.1003818)
Supplement: Table S1 — Number of embryos expressing Otx in designated blastomeres of control and morphant embryos. *1 Note that Otx was not expressed in combinations of cells not shown in this table; ‘+’ indicates the expression of Otx. *2 The expression in the a- and b-line blastomeres of the same embryos was counted separately. (DOCX) [file pgen.1003818.s009.docx]

**Table S1. Number of embryos expressing *Otx* in designated blastomeres of control and morphant embryos.**

| Expression ^*1^ | | | | Control | | Morphants | | | | | | | | | | | | |
| --- | --- | --- | --- | --- | --- | --- | --- | --- | --- | --- | --- | --- | --- | --- | --- | --- | --- | --- |
| a6.5 | a6.6 | a6.7 | a6.8 |  |  | *Fgf9/16/20* | | | *EphrinA-d* | | *Admp* | | *Gdf1/3-like* | | *Admp* &*Gdf* | | *Admp/Gdf /Fgf9/16/20* | |
| + | - | - | - | 172 | (53%) |  |  | | 8 | (19%) | 4 | (22%) | 10 | (67%) | 6 | (14%) |  |  |
| + | - | + | - | 114 | (35%) |  |  | | 10 | (23%) | 14 | (78%) | 5 | (33%) | 5 | (11%) |  |  |
| + | + | + | - |  |  |  |  | | 3 | (7%) |  |  |  |  | 9 | (20%) |  |  |
| + | + | + | + |  |  |  |  | | 2 | (5%) |  |  |  |  | 15 | (34%) |  |  |
| - | - | - | - | 41 | (13%) | 55 | (100%) | | 20 | (47%) |  |  |  |  | 9 | (20%) | 89 | (100%) |
| Total ^*2^ | | | | 327 |  | 55 |  | 43 | |  | 18 |  | 15 |  | 44 |  | 89 |  |
|  |  |  |  |  |  |  |  | |  |  |  |  |  |  |  |  |  |  |
| Expression ^*1^ | | | | Control | | Morphants | | | | | | | | | | | | |
| b6.5 | b6.6 | b6.7 | b6.8 |  |  | *Fgf9/16/20* | | | *EphrinA-d* | | *Admp* | | *Gdf1/3-like* | | *Admp* &*Gdf* | | *Admp/Gdf /Fgf9/16/20* | |
| + | - | - | - | 299 | (91%) |  |  | | 10 | (23%) | 18 | (100%) | 15 | (100%) | 10 | (23%) |  |  |
| + | + | - | - |  |  |  |  | | 11 | (26%) |  |  |  |  | 1 | (2%) |  |  |
| + | + | + | - |  |  |  |  | |  |  |  |  |  |  |  |  |  |  |
| + | + | + | + |  |  |  |  | | 4 | (9%) |  |  |  |  | 25 | (57%) |  |  |
| - | - | - | - | 28 | (9%) | 55 | (100%) | | 18 | (42%) |  |  |  |  | 8 | (18%) | 89 | (100%) |
| Total ^*2^ | | | | 327 |  | 55 |  | 43 | |  | 18 |  | 15 |  | 44 |  | 89 |  |

^*1^ Note that *Otx* was not expressed in combinations of cells not shown in this table; ‘+’ indicates the expression of *Otx*.

^*2^ The expression in the a- and b-line blastomeres of the same embryos was count separately.
