# Supplementary material for: Multiple Signaling Pathways Coordinate to Induce a Threshold Response in a Chordate Embryo
Source: PLoS Genet. 2013 Oct 3;9(10):e1003818. doi: 10.1371/journal.pgen.1003818 (PMC3789818; doi:10.1371/journal.pgen.1003818)
Supplement: Table S2 — Number of embryos expressing Nodal in designated blastomeres of control and morphant embryos. *1 Note that Nodal was not expressed in combinations of cells not shown in this table; ‘+’ indicates the expression of Nodal. *2 The expression in the a- and b-line blastomeres of the same embryos was counted separately. (DOCX) [file pgen.1003818.s010.docx]

**Table S2. Number of embryos expressing *Nodal* in designated blastomeres of control and morphant embryos.**

| Expression ^*1^ | | | | Control | | | | Morphants | | | | | | | | | | | | | | | |
| --- | --- | --- | --- | --- | --- | --- | --- | --- | --- | --- | --- | --- | --- | --- | --- | --- | --- | --- | --- | --- | --- | --- | --- |
| a6.5 | a6.6 | a6.7 | a6.8 |  |  |  |  | *Fgf9/16/20* | | *EphrinA-d* | | *Admp* | | *Gdf1/3-like* | | *Admp* &*Gdf* | | | *Admp*/*Gdf* /*Fgf9/16/20* | | | | |
| + | - | - | - |  | |  | |  |  | 1 | (2%) |  |  |  |  |  |  | |  | |  | | |
| + | - | + | - |  | |  | |  |  |  |  |  |  |  |  |  |  | |  | |  | | |
| + | + | + | - |  | |  | |  |  |  |  |  |  |  |  |  |  | |  | |  | | |
| + | + | + | + |  | |  | |  |  |  |  |  |  |  |  |  |  | |  | |  | | |
| - | - | - | - | 430 | | (100%) | | 17 | (100%) | 41 | (98%) | 14 | (100%) | 38 | (100%) | 48 | (100%) | | 46 | | (100%) | | |
| total | | | | 430 | |  | | 17 |  | 42 |  | 14 |  | 38 |  | 48 |  | 46 | |  | |  |  |
|  |  |  |  |  |  | |  |  |  |  |  |  |  |  |  |  |  |  | | |  | |  |
| Expression ^*1^ | | | | Control | | | | Morphants | | | | | | | | | | | | | | | |
| b6.5 | b6.6 | b6.7 | b6.8 |  |  |  |  | *Fgf9/16/20* | | *EphrinA-d* | | *Admp* | | *Gdf1/3-like* | | *Admp* &*Gdf* | | | *Admp/Gdf /Fgf9/16/20* | | | | |
| + | - | - | - | 404 | | (94%) | |  |  | 17 | (40%) | 14 | (100%) | 38 | (100%) | 11 | (23%) | |  | |  | | |
| + | + | - | - |  | |  | |  |  | 5 | (12%) |  |  |  |  | 9 | (19%) | |  | |  | | |
| + | + | + | - |  | |  | |  |  | 3 | (7%) |  |  |  |  | 5 | (10%) | |  | |  | | |
| + | + | + | + |  | |  | |  |  | 3 | (7%) |  |  |  |  | 4 | (8%) | |  | |  | | |
| - | - | - | - | 26 | | (6%) | | 17 | (100%) | 14 | (33%) |  |  |  |  | 19 | (40%) | | 46 | | (100%) | | |
| total | | | | 430 | | |  | 17 |  | 42 |  | 14 |  | 38 |  | 48 |  | | 46 | |  | | |

^*1^ Note that *Nodal* was not expressed in combinations of cells not shown in this table; ‘+’ indicates the expression of *Nodal*.

^*2^ The expression in the a- and b-line blastomeres of the same embryos was count separately.
